# Cefditoren and Ceftriaxone Enhance Complement-Mediated Immunity in the Presence of Specific Antibodies against Antibiotic-Resistant Pneumococcal Strains

**DOI:** 10.1371/journal.pone.0044135

**Published:** 2012-09-05

**Authors:** Elisa Ramos-Sevillano, Cinthya Rodríguez-Sosa, Fabio Cafini, Maria-Jose Giménez, Ana Navarro, David Sevillano, Luis Alou, Ernesto García, Lorenzo Aguilar, Jose Yuste

**Affiliations:** 1 Centro de Investigaciones Biológicas (CSIC) and CIBER de Enfermedades Respiratorias (CIBERES), Madrid, Spain; 2 Centro Nacional de Microbiología, Instituto de Salud Carlos III, Madrid, Spain; 3 Departamento de Microbiología, Facultad de Medicina, Universidad Complutense, Madrid, Spain; Centers for Disease Control & Prevention, United States of America

## Abstract

**Background:**

Specific antibodies mediate humoral and cellular protection against invading pathogens such as *Streptococcus pneumoniae* by activating complement mediated immunity, promoting phagocytosis and stimulating bacterial clearance. The emergence of pneumococcal strains with high levels of antibiotic resistance is of great concern worldwide and a serious threat for public health.

**Methodology/Principal Findings:**

Flow cytometry was used to determine whether complement-mediated immunity against three antibiotic-resistant *S. pneumoniae* clinical isolates is enhanced in the presence of sub-inhibitory concentrations of cefditoren and ceftriaxone. The binding of acute phase proteins such as C-reactive protein and serum amyloid P component, and of complement component C1q, to pneumococci was enhanced in the presence of serum plus either of these antibiotics. Both antibiotics therefore trigger the activation of the classical complement pathway against *S. pneumoniae*. C3b deposition was also increased in the presence of specific anti-pneumococcal antibodies and sub-inhibitory concentrations of cefditoren and ceftriaxone confirming that the presence of these antibiotics enhances complement-mediated immunity to *S. pneumoniae*.

**Conclusions/Significance:**

Using cefditoren and ceftriaxone to promote the binding of acute phase proteins and C1q to pneumococci, and to increase C3b deposition, when anti-pneumococcal antibodies are present, might help reduce the impact of antibiotic resistance in *S. pneumoniae* infections.

## Introduction

*Streptococcus pneumoniae*, the pneumococcus, is the leading bacterial cause of community-acquired pneumonia, bacteremia and meningitis, especially among young children and older adults [1]. Invasive pneumococcal disease (IPD) is associated with high morbidity and mortality, and may be fatal despite appropriate antibiotic treatment. The emergence of strains for which the minimum inhibitory concentrations (MICs) are high, complicates the success of the antibiotic treatment and poses a serious threat worldwide [2]. Immunization is a safe an highly efficient approach to preventing IPD [3]. Induction of complement immunity leads to the formation of the key component C3b that plays a major role in innate and adaptive immunity [4], [5]. Activation of the classical pathway is essential in the host immune response to *S. pneumoniae*; indeed, it is the most important route for complement activation against this pathogen in mice and humans [6], [7]. The classical pathway can also be activated by natural IgM, acute phase proteins such as C-reactive protein (CRP) and serum amyloid P component (SAP) or by the lectin SIGN-R1 [7], [8], [9], [10].

β-lactam antibiotics have little effect on the chemotaxis of neutrophils towards areas of infection. However, some can increase the oxidative burst of neutrophils by scavenging oxidative species or the inhibition of myeloperoxidase [11]. Enhancing this oxidative burst response by β-lactams may render some bacterial species more susceptible to phagocytic killing, although the mechanism is not fully understood [11], [12]. In this sense, we have previously reported that the combined effects of amoxicillin or cefotaxime with specific antibodies increases the rate of bacterial clearance in a mouse model [13]. In addition, the presence of anti-pneumococcal antibodies led to therapeutic efficacy with sub-inhibitory concentrations of β-lactam antibiotics [14]. Moreover, phagocytosis mediated by human and mouse neutrophils was increased when antibiotic-resistant pneumococcal strains were incubated with serum containing specific antibodies and sub-MIC concentrations of β-lactams [15]. Whether the synergism between anti-pneumococcal antibodies and β-lactam antibiotics is due to a direct interaction of the antimicrobial drug with the host immune system is unknown.

In the present work, three antibiotic-resistant pneumococcal strains were used to investigate the stimulation of complement-mediated immunity by sub-inhibitory concentrations of cefditoren (CDN) and ceftriaxone (CRO) in the presence of specific antibodies and serum components.

## Materials and Methods

### Ethics Statement

Healthy subjects gave their written informed consent prior to the collection of their serum. This sampling was approved by the Centro de Investigaciones Biológicas (CIB) Research Ethics Committee (Approval Reference: CIB-FJD 06010017). The immunization of animals was performed at Instituto de Salud Carlos III (ISCIII) in accordance with Spanish legislation (RD 1201/2005) and EU regulations (86/609/EEC). The animal experiments performed in this work were approved by the Animal Care and Use Committee of ISCIII (CBA PA 52_2011-v2).

### Bacterial strains and growth conditions

*S. pneumoniae* clinical isolates used in this study included strain 1515/97 [serotype 6B; penicillin (PEN) MIC = 2 µg ml^−1^; CDN MIC = 1 µg ml^−1^; CRO MIC = 2 µg ml^−1^], strain 69 (serotype 19F; PEN MIC = 2 µg ml^−1^ ml; CDN MIC = 2 µg ml^−1^; CRO MIC = 4 µg ml^−1^) and strain 48 (serotype 23F; PEN MIC = 16 µg ml^−1^; CDN MIC = 4 µg ml^−1^; CRO MIC = 8 µg ml^−1^). Pneumococcal isolates were cultured at 37°C in 5% CO_2_ on blood agar plates or in Todd-Hewitt broth supplemented with 0.5% yeast extract to an optical density at 580 nm (OD_580_) of 0.4 (approximately 10^8^ CFU ml^−1^) and stored at −70°C in 10% glycerol as single-use aliquots.

### Antibiotics and susceptibility studies

CDN was supplied from Tedec-Meiji Farma S. A. (Madrid, Spain) and CRO was purchased from Sigma-Aldrich Chemical Co. (St Louis, MO). Susceptibility was determined (in triplicate) by the agar dilution technique [16] in accordance with the criteria of the Clinical and Laboratory Standards Institute (CLSI).

### Human and mouse sera

Serum samples from five healthy male volunteers unvaccinated against *S. pneumoniae* (median age of 40 years), were obtained according to institutional guidelines and stored as single-use aliquots at −70°C to use as a source of C1q and CRP. Hyperimmune serum (HS) from mice was obtained as previously described [14], [15]. IgG levels in HS against strains 1515/97, 69 and 48 were 1056 mg ml^−1^, 371 mg ml^−1^ and 251 mg ml^−1^ respectively [15].

### Complement factors binding to *S. pneumoniae* strains

The deposition/binding of C3b, C1q and the acute phase proteins CRP and SAP to bacterial cells was assessed by flow cytometry as described previously [7], [8]. Briefly, C3b deposition was analyzed by incubating 5×10^6^ CFU of *S. pneumoniae* in 10 µl of mouse serum (diluted to 50% in PBS) for 2 h supplemented or not with 0.5 MIC and 0.25 MIC of either CDN or CRO. The bacteria were then incubated for 30 min on ice with fluorescein isothiocyanate (FITC)-conjugated polyclonal goat anti-mouse C3b antibody (ICN-Cappel) diluted 1/300 in PBS. Bacteria were then fixed in 3% paraformaldehyde and analyzed on a FACS Calibur flow cytometer (BD Biosciences) using forward and side scatter parameters to gate on at least 25,000 cells. The results were expressed as a relative % fluorescence index, providing a measure of the proportion of fluorescent bacteria positive for C3b, and the intensity of fluorescence, which quantifies bound C3b [6], [17]. Naive mouse serum, HS and HS treated with MgCl_2_ (2 mM)-EGTA (2 mM) to inhibit the activity of the lectin and classical pathways (thus allowing only alternative pathway activation), were used in C3b deposition assays [18], [19]. The interaction of C1q, CRP and SAP with *S. pneumoniae* strains in the presence or absence of both β-lactam antibiotics was evaluated following a flow cytometry method similar to that used in the C3b deposition assay. Pneumococcal strains (5×10^6^ CFU) were incubated with 10 µl of human or mouse serum (diluted to 50% in PBS) for 1 h with or without 0.5 MIC and 0.25 MIC of each antibiotic. CRP and SAP binding to the pneumococcal surface was assessed using a polyclonal rabbit anti-human CRP antibody (Calbiochem) or a polyclonal rabbit anti-mouse SAP antibody (GenScript) and a FITC-conjugated goat anti-rabbit IgG secondary antibody (Serotec), both diluted 1/300 in PBS [8]. C1q deposition was analyzed using a FITC-conjugated polyclonal sheep anti-human C1q antibody (Serotec) and a polyclonal rabbit anti-mouse C1q antibody (Abcam), followed by a secondary FITC-conjugated polyclonal goat anti rabbit (Serotec), both at a dilution of 1/300 [8].

### Statistical analysis

The data presented are the means and standard deviations for three independent experiments with at least three replicas. The results of CRP, SAP, C1q and C3b deposition assays in the presence of serum and exogenous sub-inhibitory concentrations of CDN and CRO were compared to results for control sera in the absence of each antibiotic by using the two-tailed Student's *t* test (for two groups). Analysis of variance (ANOVA) plus a post hoc Dunnett test was used for multiple comparisons. GraphPad InStat version 3.0 (GraphPad Software, San Diego, CA) was used for statistical analysis.

## Results

### Interaction of acute phase proteins with antibiotic-resistant pneumococcal strains in the presence of CDN or CRO

**Figure 1:**
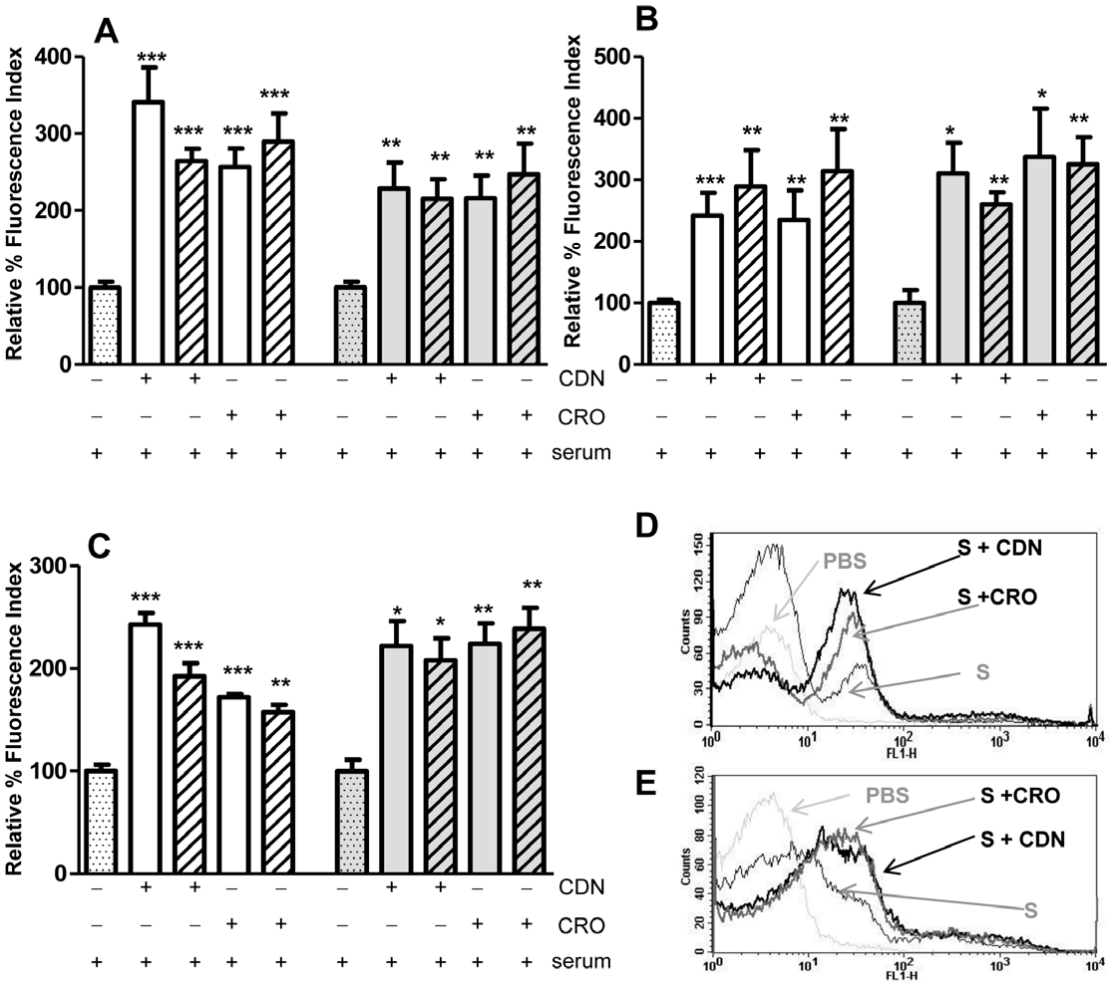
Binding of acute phase proteins to the different bacterial strains in the presence or absence of CDN or CRO. Deposition of human CRP (white bars) and mouse SAP (grey bars) in the presence of serum alone (dotted bars) or serum with 0.5 MIC (open bars) and 0.25 MIC (hatched bars) of either cefditoren (CDN) or ceftriaxone (CRO). (A) Deposition of CRP and SAP on the surface of the serotype 23F strain. (B) Deposition of CRP and SAP on the surface of the serotype 19F strain. (C) Deposition of CRP and SAP on the surface of the serotype 6B strain. (D) Example of a flow cytometry histogram for CRP binding on the serotype 23F strain. (E) Example of a flow cytometry histogram for SAP binding on the 6B strain. Results are expressed as relative % fluorescence indices relative to the results for sera in the absence of antibiotics. Error bars represent standard deviations; asterisks mark statistically significant differences with sera in the absence of antibiotics (two-tailed Student *t* test *P<0.05, **P<0.01, ***P<0.001). ***P<0.001 for serotypes 23F and 6B and * *P*<0.05 for serotype 19F in the multiple comparison of CRP binding (one-way ANOVA with a post hoc Dunnet test). * *P*<0.05 for serotypes 23F and 19F and ** *P*<0.01 for serotype 6B in the multiple comparison of SAP binding (one-way ANOVA with a post hoc Dunnet test).

The recognition of the different *S. pneumoniae* strains by human CRP and mouse SAP was investigated by flow cytometry in the presence or absence of sub-inhibitory concentrations of CDN or CRO. For all the strains, and with both antibiotics, the binding of human CRP and mouse SAP was significantly increased (Fig. 1). These results suggest that recognition of antibiotic-resistant *S. pneumoniae* strains by acute phase proteins such as CRP and SAP is enhanced when bacteria are exposed to these antibiotics.

### Activation of the classical complement pathway against antibiotic-resistant *S. pneumoniae* strains by CDN and CRO

**Figure 2:**
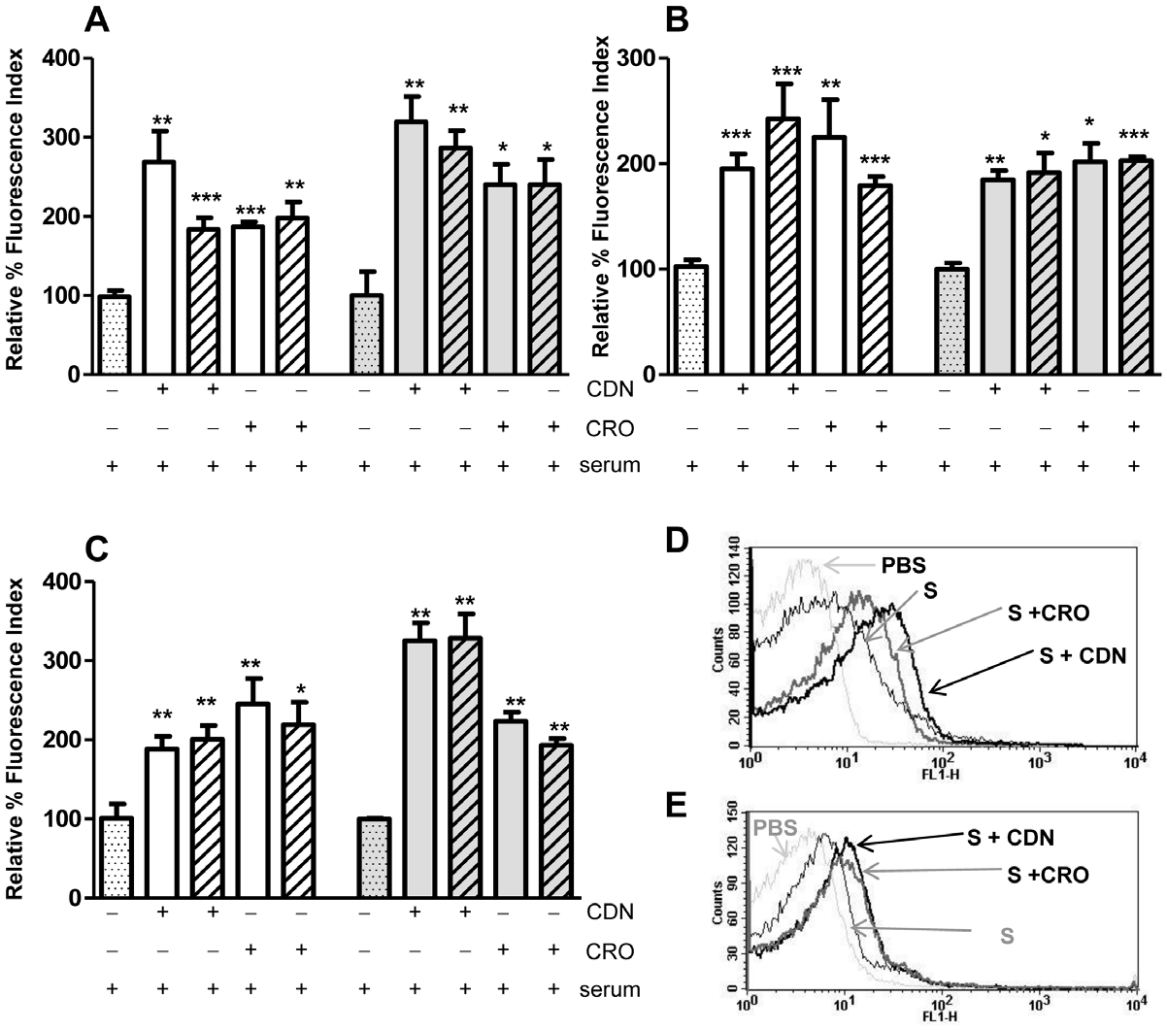
C1q deposition in the presence or absence of CDN or CRO. Deposition of human C1q (white bars) or mouse C1q (grey bars) using serum alone (dotted bars) or serum with 0.5 MIC (open bars) and 0.25 MIC (hatched bars) of either CDN or CRO. (A) Deposition of C1q on the surface of the serotype 23F strain. (B) Deposition of C1q on the surface of the serotype 19F strain. (C) Deposition of C1q on the surface of the serotype 6B strain. (D) Example of a flow cytometry histogram for human C1q binding on the 23F strain. (E) Example of a flow cytometry histogram for mouse C1q on the 19F strain. Results are expressed as relative % fluorescence indices relative to the results for sera in the absence of antibiotics. Error bars represent standard deviations; asterisks mark results that are statistically significant compared to those for sera with no antibiotics (two-tailed Student *t* test *P<0.05, **P<0.01, ***P<0.001). ***P<0.001 for strains of serotypes 23F and 19F and * *P*<0.05 for 6B strain in the multiple comparison of human C1q binding (one-way ANOVA with a post hoc Dunnet test). **P<0.001 for serotypes 23F and 6B and * *P*<0.05 for serotype 19F in the multiple comparison of mouse C1q binding (one-way ANOVA with a post hoc Dunnet test).

To investigate the activation of the classical pathway in the presence of the antibiotics, pneumococcal resistant strains were incubated with human or mouse serum in the presence or absence of sub-inhibitory concentrations of CDN or CRO, and the deposition of either human or mouse C1q was analyzed. Bacteria incubated with human or mouse sera supplemented with 0.5 MIC and 0.25 MIC of either CDN or CRO showed greater levels of C1q bound to the bacterial surface than those incubated in serum alone (Fig. 2). These results confirm that the capacity of C1q to recognize *S. pneumoniae* and further stimulate the classical complement pathway is enhanced in the presence of these antibiotics (Fig. 2).

### C3b deposition mediated by serum containing anti-pneumococcal antibodies and sub-inhibitory concentrations of CDN or CRO

**Figure 3:**
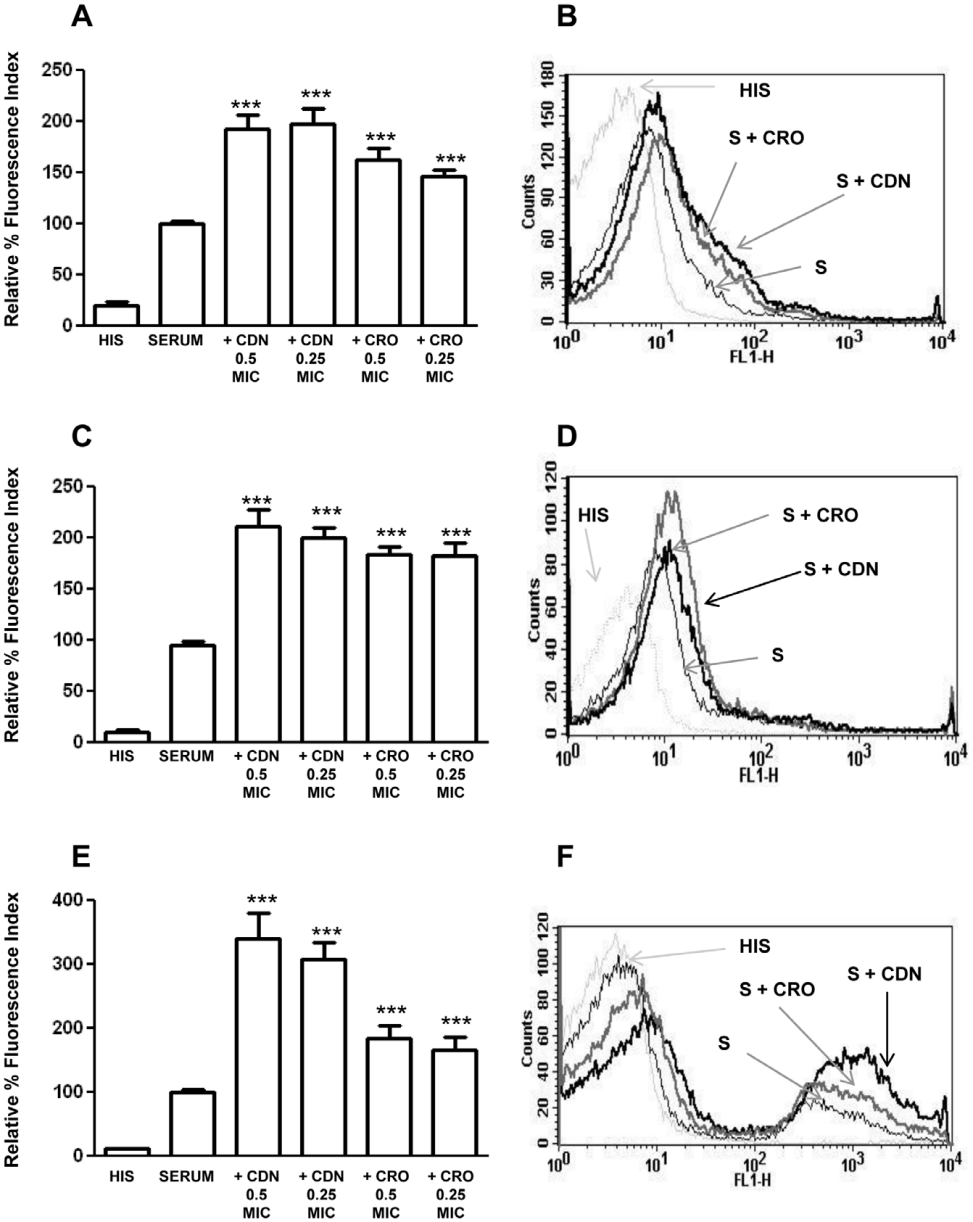
C3b deposition on the surface of the different bacterial strains using hyperimmune serum containing anti-pneumococcal antibodies in the presence or absence of 0.5 MIC and 0.25 MIC of CDN or CRO. (A) Deposition of C3b on the surface of the serotype 23F strain. (B) Example of a flow cytometry histogram for C3b deposition on the serotype 23F strain. (C) Deposition of C3b on the serotype 19F strain. (D) Example of a flow cytometry histogram for C3b binding on the serotype 19F strain. (E) Deposition of C3b on the surface of the serotype 6B strain. (F) Example of a flow cytometry histogram for C3b binding on the serotype 6B strain. Results are expressed as relative % fluorescence indices relative to the results for sera in the absence of antibiotics. Error bars represent standard deviations; asterisks mark results that are statistically significant compared to those for sera in the absence of antibiotics (two-tailed Student *t* test *P<0.05, **P<0.01, ***P<0.001). ***P<0.001 for all the strains in the multiple comparison of C3b binding (one-way ANOVA with a post hoc Dunnet test).

Pneumococcal isolates were incubated in sera from immunized mice in the presence or absence of sub-inhibitory concentrations of CDN and CRO or in PBS and serum heated to 65°C for 30 min (heat inactivated serum; HIS) as negative controls (Fig. 3). HIS conserves antibody function but lacks any complement activity, demonstrating the specificity of the assay for detecting functional C3b. No C3b deposition was detected on pneumococcal strains incubated in PBS or HIS, (Fig. 3). Opsonization of the different strains with the corresponding HS and 0.5 MIC or 0.25 MIC of either CDN or CRO significantly increased C3b deposition compared to that seen on bacteria incubated in serum alone (Fig. 3). Strain 1515/97 showed the strongest C3b deposition in the presence of the antibiotics (Figs. 3E and F). Overall, this C3b deposition was improved when bacteria were opsonized with serum containing anti-pneumococcal antibodies in the presence of sub-inhibitory concentrations of CDN or CRO, although deposition was significantly greater in the presence of CDN (*P*<0.05, two-tailed *t* test) for the serotype 6B strain (Figs. 3E and F).

**Figure 4:**
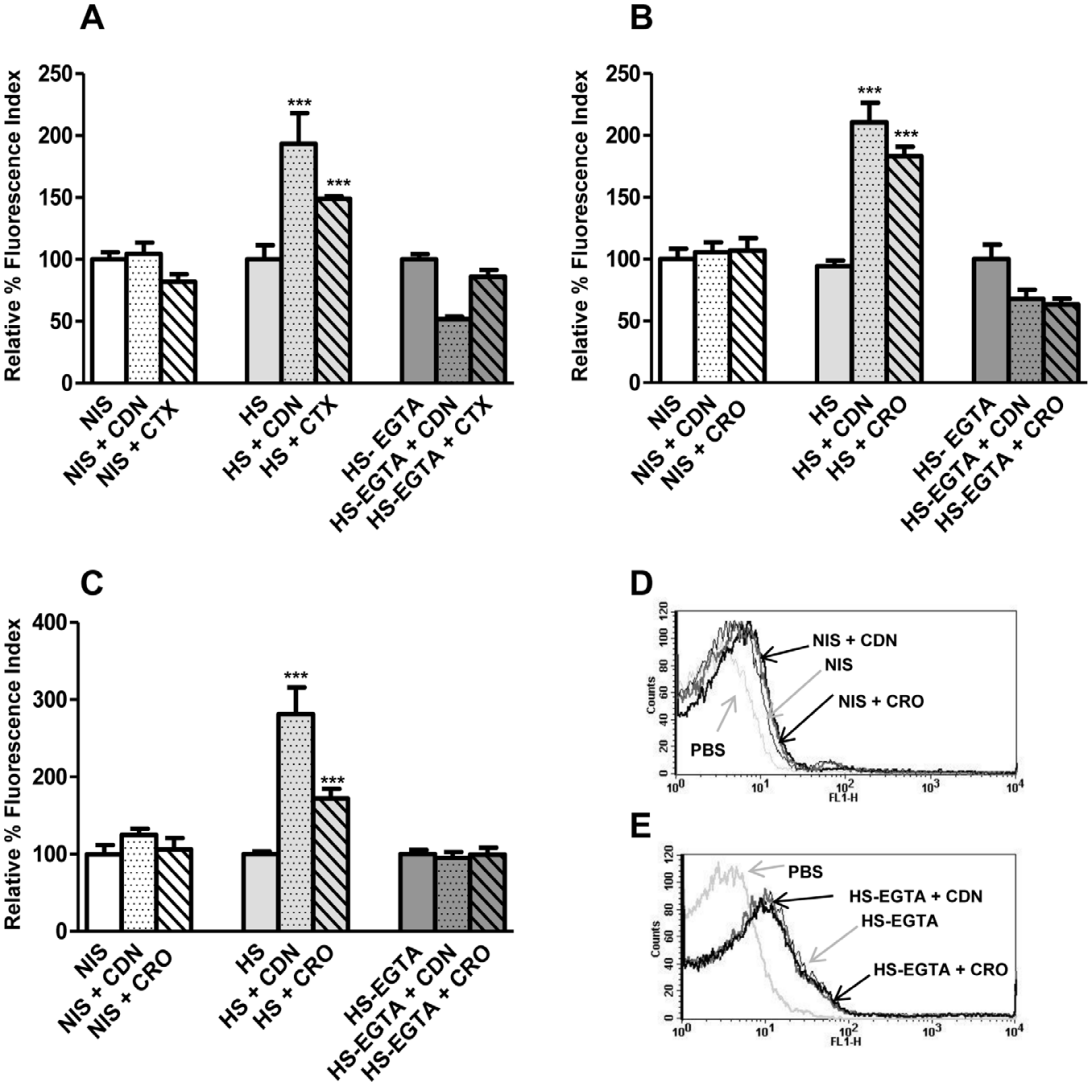
C3b deposition on the different bacterial strains using non-immune mouse serum (NIS), hyperimmune mouse serum (HS) and HS treated with EGTA (HS-EGTA). Deposition of C3b was measured using NIS (white bars), HS (light grey bars) or HS-EGTA (dark grey bars) in the absence or presence of 0.5 MIC CFD or CRO. (**A**) Deposition of C3b on the surface of the serotype 23F strain. (B) Deposition of C3b on the surface of the serotype 19F strain. (C) Deposition of C3b on the surface of the serotype 6B strain. (D) Example of a flow cytometry histogram for C3b binding on the serotype 6B strain incubated with NIS. (E) Example of a flow cytometry histogram for C3b binding on the serotype 6B strain incubated with HS-EGTA. Results are expressed as relative % fluorescence indices relative to the results for sera in the absence of antibiotics. Error bars represent standard deviations; asterisks mark results that are statistically significant compared to those for sera in the absence of antibiotics (two-tailed Student *t* test, ***P<0.001).

To demonstrate the importance of antibodies in the increased C3b deposition mediated by CDN or CRO, bacteria were incubated using non-immune mouse serum (NIS) and the results were compared with those obtained with HS. The results confirmed the antibodies to be necessary for the antibiotic-enhanced C3b deposition (Fig. 4). Activation of the alternative complement pathway by the antibiotics was also investigated. HS was treated with a mixture of magnesium-EGTA to chelate the calcium ions needed for classical and lectin pathway activation; the only functional cascade left was, therefore, the alternative pathway. Loss of classical pathway activity prevented any enhancement of C3b deposition in the presence of the antibiotics. This confirms that the increased recognition of *S. pneumoniae* by the complement system in the presence of sub-inhibitory concentrations of CDN or CRO is mediated by the activation of the classical pathway (Fig. 4).

## Discussion

IPD occurs when colonizing microorganisms in the nasopharynx translocate to alveolar spaces and bloodstream, causing bacteraemic pneumonia and sepsis, or when they traverse the blood-brain barrier, causing meningitis [20]. In the absence of antibiotic treatment, the clinical outcome depends entirely on the interactions between bacterial virulence factors and the host defense mechanisms. Clearance of pneumococci from the systemic circulation strongly depends on opsonization by complement components and phagocytosis [21], [22]. PEN-resistant *S. pneumoniae* strains have a peptidoglycan structure containing more hydrophobic peptides (carrying dialanyl or alanylserine cross bridges) than PEN-susceptible isolates [23]. Muramyl dipeptide, the breakdown product of bacterial cell walls from PEN-treated pneumococci, is a powerful stimulator of the immune response. The impaired released of this cell wall component in resistant strains might be involved in the reduced immunogenicity of capsular serotypes usually associated with PEN-resistance [24], [25]. In this sense, HS against the serotype 6B strain (that had the lowest MIC to CDN and CRO among the three strains used) contained the higher antibody titer compared to HS against the 19F and 23F strains [15].

It is widely accepted that β-lactam antibiotics exert their bactericidal activity by a direct effect of the antimicrobial agent on the target microorganism. However, IPD is associated to high rates of morbidity and mortality worldwide despite proper antibiotic therapy [24]. This common lack of antibiotic efficacy is especially evident in immunocompromised patients, suggesting that the recovery of these patients depends on the successful joint action of antibiotics and host defense mechanisms. The emergence of pneumococcal strains strongly resistant to antibiotics could therefore jeopardize the success of the antibiotic therapy [2]. Alteration of cell surface structures by antibiotics might result in greater exposure of deeper antigenic epitopes that are normally hidden or hardly exposed. Such exposure may promote opsonization by several host defense components such as acute phase proteins, enhancing the recognition of the microbial pathogen by professional phagocytes. In this sense, the use of cephalosporins has been linked to an increased bactericidal serum activity against *Escherichia coli* and *Pseudomonas aeruginosa* [26], [27]. Alternatively, antibiotics might reduce the expression of virulence factors involved in inhibition of complement activation. Indeed, a recent report has confirmed that certain antibiotics at sub-inhibitory concentrations modify virulence gene expression in *S. aureus* [28].

CRP and SAP belong to the pentraxin family and are the main acute phase proteins in humans and mice, respectively [8], [29], [30], [31]. CRP levels rise dramatically during pneumococcal infection highlighting the importance of this acute phase protein as a sentinel molecule against this pathogen [30]. One of the main roles played by CRP and SAP in host defense against pneumococci is the opsonization of the microorganism and induction of phagocytosis via Fcγ-receptors [8], [32], [33]. The present results show that the recognition of *S. pneumoniae* by CRP and SAP was enhanced when bacteria were opsonized with serum in the presence of sub-inhibitory concentrations of CDN or CRO suggesting that these antibiotics allow CRP and SAP to recognize *S. pneumoniae* more efficiently. This agrees with the findings of a recent study in which we detected increased phagocytosis (both by human and mouse neutrophils) of *S. pneumoniae* when opsonized with serum containing specific anti-pneumococcal antibodies and sub-inhibitory concentrations of either CDN or CRO [15]. Pentraxin 3 has also been shown to be very effective in combination to antimycotic drugs against *Aspergillus fumigatus* infections, stimulating the antifungal activity of effector phagocytes [34]. Increased damage of the microbial surface by antimicrobial drugs might allow certain bacterial components of the cell envelope to become more accessible to complement components and antibodies. Exposure of *Klebsiella pneumoniae* strains to serum complement and β-lactam antibiotics increased C3 levels on the bacterial surface [35].

In complement-mediated defense against *S. pneumoniae*, the classical pathway is triggered by the first component (C1q) which directly recognizes certain ligands on the microbial surface or can be activated by acute phase proteins and specific immunoglobulins [7], [36]. The present results showed that C1q deposition on the bacterial surface was increased in the presence of the studied antibiotics, confirming that bacterial alterations caused by CDN and CRO enhance complement-mediated immunity to *S. pneumoniae* via a mechanism dependent on classical pathway activation.

Once the complement cascade is activated, the central complement component C3b is formed by a complex enzymatic system [4], [5]. C3b deposition on the bacterial surface was significantly increased when bacteria were opsonized with serum and sub-MIC levels of CDN or CRO. Certainly, anti-pneumococcal antibodies can modify the bacteraemic profile and delay the course of IPD in mice models [37], [38], and indeed, the present results obtained when using NIS show that specific antibodies are important for the enhanced C3b deposition. A previous study involving the same strains showed that bacterial clearance and survival rates mediated by anti-pneumococcal antibodies were greatly improved after the administration of a sub-therapeutic dose of either CDN or CRO [15]. These findings mirror the results of the present complement immunity assays, and demonstrate that the collaboration of complement-mediated immunity (via the classical pathway) and β-lactam therapy for IPD favours phagocytosis.

Early antibiotic treatment is essential to prevent severe morbidity and mortality because delays in treatment increase the chances of disability and death. This problem becomes worse when the invading pathogen harbors resistance to antibiotics. When faced with highly resistant pneumococcal strains, treatment with CFD or CRO may be a novel strategy to reduce the risk of treatment failure in people previously vaccinated against *S. pneumoniae* by enhancing the efficiency of the complement-mediated immune response.
